# Optimized Extraction, Identification and Anti-Biofilm Action of Wu Wei Zi (*Fructus Schisandrae Chinensis*) Extracts against *Vibrio parahaemolyticus*

**DOI:** 10.3390/molecules28052268

**Published:** 2023-02-28

**Authors:** Zongyi Zhang, Yanan Zhao, Jing Cai, Tong Wang, Yujie Song, Jingyi Lu, Hairuo Du, Wenfang Wang, Yan Zhao, Lei Guo

**Affiliations:** 1 Jiangsu Key Laboratory of Marine Bioresources and Environment, Co-Innovation Center of Jiangsu Marine Bio-Industry Technology, Jiangsu Ocean University, Lianyungang 222005, China; 2 Jiangsu Key Laboratory of Marine Biotechnology, Jiangsu Ocean University, Lianyungang 222005, China

**Keywords:** Wu Wei Zi, *Vibrio parahaemolyticus*, antibacterial, anti-biofilm, extraction optimization

## Abstract

The pathogenicity of foodborne *Vibrio parahaemolyticus* is a major concern for global public health. This study aimed to optimize the liquid–solid extraction of Wu Wei Zi extracts (WWZE) against *Vibrio parahaemolyticus*, identify its main components, and investigate the anti-biofilm action. The extraction conditions optimized by the single-factor test and response surface methodology were ethanol concentration of 69%, temperature at 91 °C, time of 143 min, and liquid–solid ratio of 20:1 mL/g. After high performance liquid chromatography (HPLC) analysis, it was found that the main active ingredients of WWZE were schisandrol A, schisandrol B, schisantherin A, schisanhenol, and schisandrin A–C. The minimum inhibitory concentration (MIC) of WWZE, schisantherin A, and schisandrol B measured by broth microdilution assay was 1.25, 0.625, and 1.25 mg/mL, respectively, while the MIC of the other five compounds was higher than 2.5 mg/mL, indicating that schisantherin A and schizandrol B were the main antibacterial components of WWZE. Crystal violet, Coomassie brilliant blue, Congo red plate, spectrophotometry, and Cell Counting Kit-8 (CCK-8) assays were used to evaluate the effect of WWZE on the biofilm of *V. parahaemolyticus*. The results showed that WWZE could exert its dose-dependent potential to effectively inhibit the formation of *V. parahaemolyticus* biofilm and clear mature biofilm by significantly destroying the cell membrane integrity of *V. parahaemolyticus*, inhibiting the synthesis of intercellular polysaccharide adhesin (PIA), extracellular DNA secretion, and reducing the metabolic activity of biofilm. This study reported for the first time the favorable anti-biofilm effect of WWZE against *V. parahaemolyticus*, which provides a basis for deepening the application of WWZE in the preservation of aquatic products.

## 1. Introduction

*Vibrio parahaemolyticus* is a common foodborne pathogen that can infect any animal, including humans [1,2]. This gram-negative halophilic bacterium is ubiquitous in estuaries and coastal waters, causing varying degrees of pollution to marine fish, mollusks, and crustaceans. Eating raw, undercooked, or improperly handled seafood can cause acute gastroenteritis, causing abdominal pain, diarrhea, nausea, vomiting, and other symptoms [3,4,5,6]. In regions such as the United States, China, Japan, and South Korea, *V. parahaemolyticus* has become a major cause of seafood-related gastroenteritis, posing a serious threat to public health [7]. Although traditional antibiotics and chemical bacteriostatic agents are highly effective, long-term overuse and abuse cause some bacteria to develop drug resistance, posing a threat to human health [8]. Therefore, green, harmless, non-toxic, and effective bacteriostatic substances should be used before food is consumed to inhibit the growth of harmful pathogenic bacteria in shelf life.

Bacterial biofilms are complex microbial communities attached to biological or abiotic surfaces that protect them against different hostile conditions such as disinfectants, antibiotics, and other hygienic conditions by providing a robust three-dimensional, multicellular, complex, self-assembled extracellular polymeric structure [9]. Another key problem in biofilm formation is their antibiotic resistance, which makes drug treatment difficult [10,11]. Biofilms are often persistent in food processing environments as a source of cross-contamination and foodborne illness, and have been reported to be associated with more than 80% of bacterial infections and approximately 60% of foodborne outbreaks in the population [12]. Therefore, there is an urgent need to develop effective and robust strategies to control the formation of pathogen biofilms. Many natural products with biofilm inhibitory activity have been identified, including ellagic acid glycosides, hamamelitannin, carolacton, skyllamycins, promysalin, phenazines, bromoageliferin, flustramine C, meridianin D, and brominated furanones [13]. Furthermore, some synthesized derivatives have reported promising anti-biofilm properties in clinically relevant Gram-positive and Gram-negative pathogens [14,15].

Wu Wei Zi, as a natural active drug, has the advantages of abundant resources, low price, high safety, low toxicity, and low resistance compared with chemical synthetic drugs, and is a high-quality resource for the development of alternatives to chemical synthetic antibacterial agents [16,17]. Wu Wei Zi, also called *Fructus Schisandrae Chinensis*, Chinese magnolia-vine fruit, or Bei Wu Wei Zi, refers to the dried ripe fruit of the Magnoliaceae *Schisandra chinensis* (Turcz.) Baill. [18]. Wu Wei Zi has been used for treating cough, asthma, insomnia, dreaminess, and other nervous system and kidney related diseases in China and other East Asian countries for thousands of years [19]. Modern pharmacological researches have indicated that it has anti-cancer, anti-microbial, anti-diabetic, anti-aging, anti-obesity, and other biological activities, and ligands were the main active ingredients [20,21].

In our previous study, Wu Wei Zi was found to have favorable anti-*V. parahaemolyticus* properties. Based on this, this study was conducted to optimize the liquid–solid extraction process, identify the main antibacterial components, and investigate the anti-biofilm activity and mechanism of action of Wu Wei Zi extracts (WWZE) against *V. parahaemolyticus*.

## 2. Results

### 2.1. Effect of Single Factor on Antibacterial Activity of WWZE against V. parahaemolyticus

**Figure 1:**
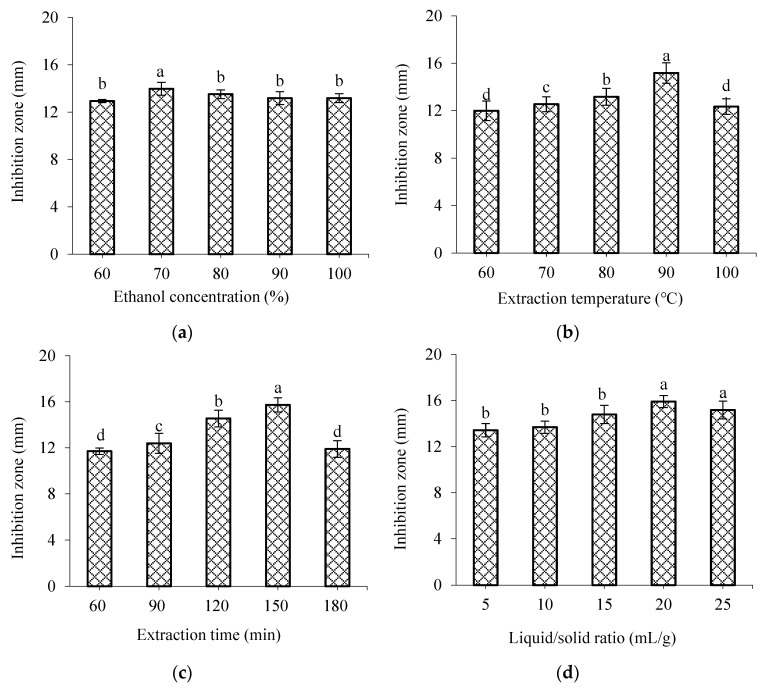
Effects of different factors on the anti-*V. parahaemolyticus* activity of WWZE. (**a**) Ethanol concentration; (**b**) Extraction temperature; (**c**) Extraction time; (**d**) Liquid/solid ratio. Different lowercase letters represent significant differences (*p* < 0.05).

The influence of different factors on the anti-*V. parahaemolyticus* activity of WWZE is shown in Figure 1. As can be seen from Figure 1a, when the ethanol concentration increased from 60% to 70%, the antibacterial effect of WWZE on *V. parahaemolyticus* increased. When the proportion of ethanol increased further, the antibacterial effect was no longer improved. As can be seen from Figure 1b, when the extraction temperature was 60–90 °C, the antibacterial effect of WWZE increased with the increase of temperature. However, excessive temperature may degrade the antibacterial active substances, showing that the antibacterial effect of WWZE decreased at 100 °C. As can be seen from Figure 1c, the antibacterial effect of WWZE on *V. parahaemolyticus* significantly increased when extraction time was 60–150 min, but continued extension of extraction time had no further positive effect on antibacterial activity of WWZE. As can be seen from Figure 1d, with the increase of liquid–solid ratio, the antibacterial effect of WWZE on *V. parahaemolyticus* also increased, and reached the maximum at 20:1 mL/g.

### 2.2. Optimization of Extraction Process

In order to save the extraction solvent and reduce the difficulty of subsequent treatment, the liquid–solid ratio was fixed at 20:1 mL/g, and ethanol concentration of 70%, extraction temperature of 90 °C, and extraction time of 150 min were selected as the central points of the three independent variables of Box–Behnken Design (BBD). The diameter of the inhibitory circle of WWZE against *V. parahaemolyticus* was taken as the response variable (*Y*, mm). A total of 17 experiments with three factors and three levels were performed. The design scheme and its results are shown in Table 1.

By applying multiple regression analysis of the experimental data, the equation between the diameter of inhibitory circle and independent variables was established as follows:

*Y* = 15.34 − 0.028*X*_1_ + 0.32*X*_2_ − 0.44*X*_3_ − 0.64*X*_1_*X*_2_ + 0.43*X*_1_*X*_3_ − 0.49*X*_2_*X*_3_ − 1.22*X*_1_^2^ − 1.73*X*_2_^2^ − 1.14*X*_3_^2^

Table 2 shows the variance analysis of the effects of ethanol concentration, extraction temperature, and extraction time on the diameter of inhibitory circle of WWZE. As can be seen from Table 2, the coefficient of determination (*R*^2^) was 0.9723, indicating the model sufficiently represented the real relationship between the independent and response variables. The *p* value (0.0001) and the value of lack of fit (0.1001) indicated that the suitability of the model was significant. The effects of extraction temperature and extraction time were significant (*p* < 0.05). The interaction effect between ethanol concentration and extraction temperature was very significant (*p* < 0.01), indicating that there was not a simple linear relationship between ethanol concentration and extraction temperature, but also significant interaction between ethanol concentration and extraction time, extraction temperature, and extraction time (*p* < 0.05). The secondary effects of ethanol concentration, extraction temperature, and extraction time were significant (*p* < 0.01).

**Figure 2:**
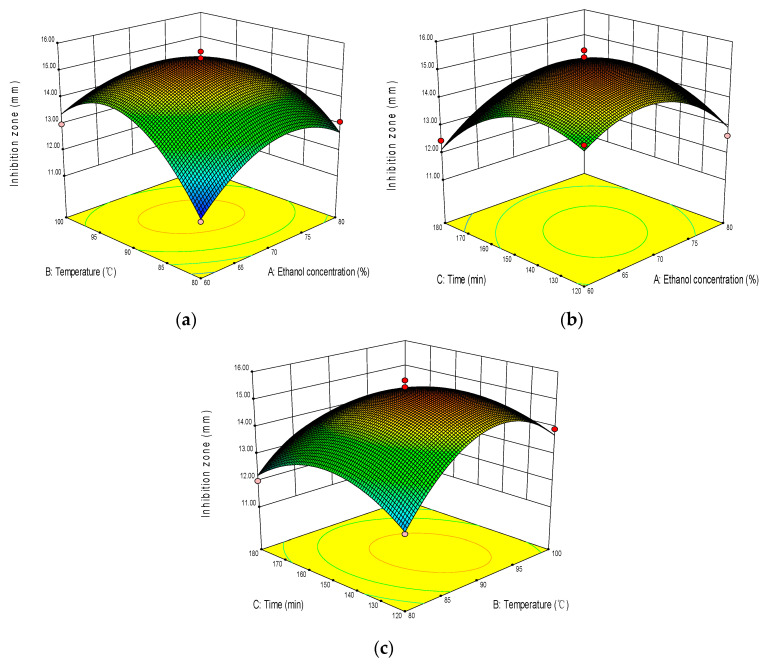
Response surface analysis plots of the interaction effects of (**a**) ethanol concentration and temperature, (**b**) ethanol concentration and time, and (**c**) temperature and time.

The response surface analysis of the interaction effects of ethanol concentration, extraction temperature, and extraction time is shown in Figure 2. Figure 2 shows that the response surface is a convex surface with a downward opening, and the center of the response surface is located in the investigated area, indicating that there is the maximum interaction of all influencing factors within the selected range. In the study range, with the increase of ethanol concentration, extraction temperature, and time, the diameter of inhibitory circle of WWZE against *V. parahaemolyticus* all showed a trend of first increasing and then decreasing. The optimal extraction parameters were predicted by regression model as *X*_1_ = 69%, *X*_2_ = 91 °C, and *X*_3_ = 143 min. The average diameter of inhibitory circle was 15.67 ± 0.13 mm, but there was no significant difference to predicted values (15.42 mm), indicating that RSM had good feasibility to optimize the extraction process of WWZE. Subsequently, the extracts were vacuum rotary evaporated and dried to obtain solid WWZE, the yield of which was 24.53 ± 0.67%.

### 2.3. Identification of Anti-V. Parahaemolyticus Active Ingredients in WWZE

**Figure 3:**
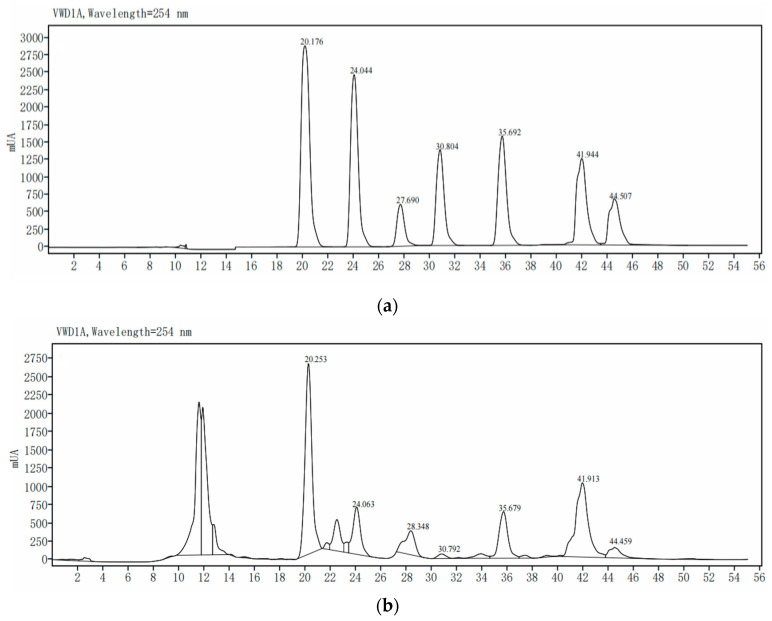
HPLC chromatogram of standard mixtures (**a**) and WWZE (**b**).

Figure 3 shows the HPLC chromatograms of the standard mixtures and WWZE; the retention times (t_R_) of schisandrol A, schisandrol B, schisantherin A, schisanhenol, schisandrin A, schisandrin B, schisandrin C in the standard mixtures were as follows: 20.176, 24.044, 27.690, 30.804, 35.692, 41.944, and 44.507 min (Figure 3a). As can be seen from Figure 3b, WWZE contained a variety of chemical components including the above seven compounds, and the corresponding retention time (t_R_) was 20.253, 24.063, 28.348, 30.792, 35.679, 41.913, and 44.459 min, respectively. The content of schisandrol A, schisandrol B, schisantherin A, schisanhenol, schisandrin A, schisandrin B, schisandrin C in WWZE was 2.59, 0.746, 1.55, 0.129, 1.157, 2.34, and 0.614 mg/g, respectively.

The broth microdilution method was used to determine the anti-*V. parahaemolyticus* activities of WWZE and its seven chemical components. The MIC values of WWZE, schisantherin A, and schisandrol B determined were 1.25, 0.625, and 1.25 mg/mL, respectively, while the MIC value of the other five compounds was greater than 2.5 mg/mL, indicating that schisantherin A and schizandrol B were the main antibacterial components of WWZE.

### 2.4. Antibiofilm Activity of WWZE against V. parahaemolyticus

**Figure 4:**
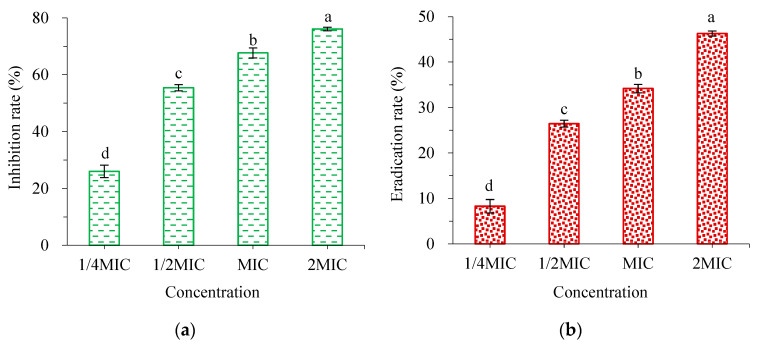
Antibiofilm activity of WWZE against *V. parahaemolyticus*. (**a**) Biofilm formation inhibition; (**b**) mature biofilm eradication. Different lowercase letters represented significant differences (*p* < 0.05) and WWZE.

As shown in Figure 4a, the inhibitory effect of WWZE on the biofilm formation of *V. parahaemolyticus* increased with the increase of concentration, and the inhibitory rate was greater than 50% at 0.5MIC and 76.12% at 2MIC. As can be seen from Figure 4b, the clearance effect of WWZE on the mature biofilm of *V. parahaemolyticus* also showed a dose-dependent pattern, with the eradication rate of 26.44% and 46.28% at 0.5MIC and 2MIC, respectively, which were far lower than the inhibition rate at the same concentration.

### 2.5. Antibiofilm Mechanism of Action of WWZE against V. parahaemolyticus

#### 2.5.1. Effect of WWZE on the Membrane Integrity of *V. parahaemolyticus*

**Figure 5:**
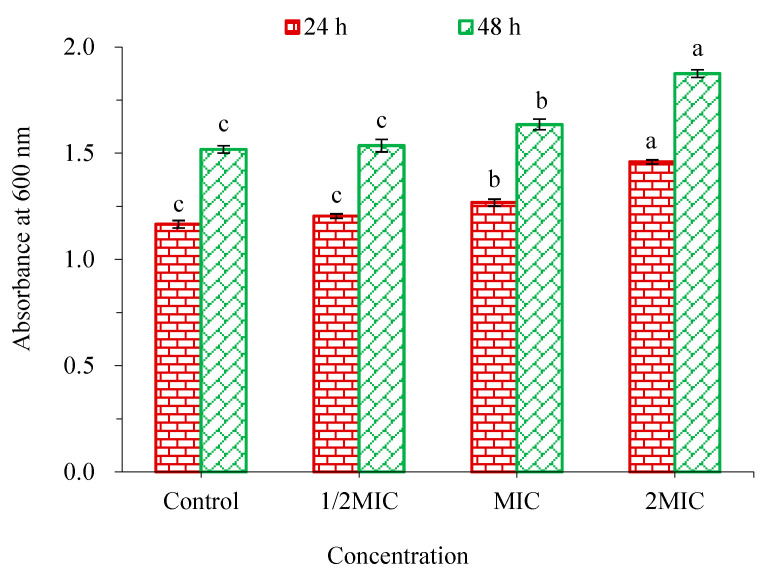
Effect of WWZE on the membrane integrity of *V. parahaemolyticus* during biofilm formation (24 h) and matured biofilm (48 h). Different lowercase letters indicated significant differences (*p* < 0.05).

The effect of WWZE on the membrane integrity of *V. parahaemolyticus* was characterized by Coomassie brilliant blue method by measuring the extracellular protein content (Figure 5). It can be seen that when the concentration of WWZE reaches MIC, the extracellular protein content of the bacteria increased significantly, whether in the forming biofilm or mature biofilm. The results showed that the membrane integrity of *V. parahaemolyticus* was damaged after treatment with WWZE, which resulted in the leakage of intracellular proteins outside the cell.

#### 2.5.2. Effect of WWZE on Polysaccharide Intercellular Adhesin (PIA) Synthesis during Biofilm Formation of *V. parahaemolyticus*

**Figure 6:**
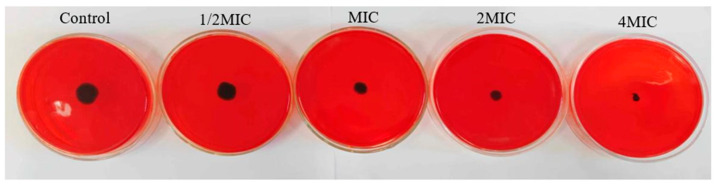
Effect of WWZE on PIA synthesis during biofilm formation of *V. parahaemolyticus*.

Congo red can react with the surface PIA of the bacterial biofilms to darken the colony. It can be seen from Figure 6 that the colony of *V. parahaemolyticus* showed black in the control group without WWZE treatment, while the black colonies of *V. parahaemolyticus* gradually became smaller in the WWZE-treated groups with the increase of the concentration of WWZE. When the concentration of WWZE reached MIC, the colony diameter visually showed a significant decrease. At 4 MIC, the colony became very small, indicating that WWZE can inhibit the synthesis of PIA during the biofilm formation of *V. parahaemolyticus*, and with the increase of the concentration of WWZE, the stronger the inhibitory effect on PIA synthesis during the biofilm formation of *V. parahaemolyticus*.

#### 2.5.3. Effects of WWZE on Extracellular DNA (eDNA) Secretion during Biofilm Formation of *V. parahaemolyticus*

**Figure 7:**
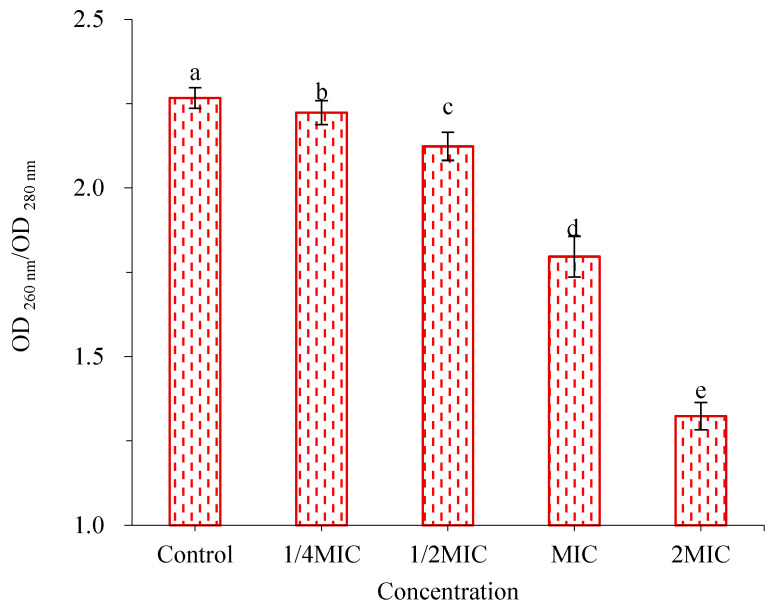
Effect of WWZE on eDNA secretion during biofilm formation of *V. parahaemolyticus*. Different lowercase letters indicated significant differences (*p* < 0.05).

As can be seen from Figure 7, the eDNA secretion of *V. parahaemolyticus* gradually decreased with the increase concentration of WWZE. Compared with the control group, WWZE at 0.25MIC concentration significantly reduced the eDNA secretion of *V. parahaemolyticus*, which was reduced by 58.38% (*p* < 0.01) at 2MIC concentration, indicating that WWZE could inhibit the eDNA secretion of *V. parahaemolyticus*, and the degree of inhibition increased with the increase of WWZE concentration.

#### 2.5.4. Effect of WWZE on Biofilm Metabolic Activity of *V. parahaemolyticus*

**Figure 8:**
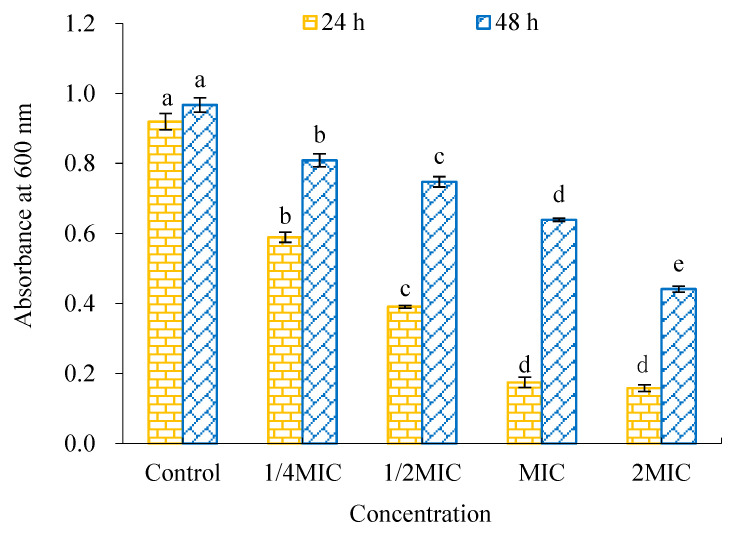
Effect of WWZE on the metabolic activity of *V. parahaemolyticus* during biofilm formation (24 h) and matured biofilm (48 h). Different lowercase letters indicate significant differences (*p* < 0.05).

As shown in Figure 8, the metabolic activity of *V. parahaemolyticus* biofilm decreased more significantly with the increase concentration of WWZE. Compared with the control group, 0.25MIC concentration of WWZE significantly decreased the metabolic activity of both forming and mature biofilms (*p* < 0.05). The metabolic activity of forming and mature biofilms was reduced to 19.02% and 66.08%, respectively, by MIC concentration of WWZE. The results showed that WWZE not only had significant inhibition on the metabolic activity in the process of biofilm formation, but also had a certain inhibitory effect on the metabolic activity of mature biofilm.

## 3. Discussion

As a homologous resource of food and medicine, Wu Wei Zi shows good application potential in pharmaceutical, cosmetic, and food industries based on its extensive pharmacological activities, especially antibacterial properties. Song et al. found that in the barley soup model food system, the aqueous extract of Wu Wei Zi showed the high antibacterial activity against *Staphylococcus aureus* and significantly reduced total viable bacterial counts, showing potential in preventing the growth of foodborne pathogens [22]. Furthermore, Cui et al. found that in model cosmetic system of O/W emulsions, the aqueous extract of Wu Wei Zi has great bacteriostatic activity and multi-targets on *Escherichia coli* with MBC of 18 mg/mL, which can be used as a candidate cosmetic preservative [23]. Bai et al. studied the activity of ethanol extract and aqueous extract of Wu Wei Zi against typical foodborne pathogens and food spoilage microorganisms. Both extracts have significant antimicrobial activities against *Staphylococcus aureus*, *Listeria monocytogenes*, *Bacillus subtilis*, *Pseudomonas aeruginosa*, and *Escherichia coli* [24]. Up to now, the extraction process of antibacterial substances against *V. parahaemolyticus* of Wu Wei Zi has not been reported.

Considering that this study aimed to develop food preservatives, it was necessary to use eco-friendly solvents to extract antibacterial active substances [17,25]. Therefore, this study investigated the liquid–solid extraction process of WWZE with ethanol solution as the solvent, and the results showed that the optimal ethanol concentration was 69%, indicating that the anti-*V. parahaemolyticus* active components of Wu Wei Zi contain both hydrophilic and hydrophobic components [18]. This is similar to the results of Bai et al. [24], who found that the ethanol extract of Wu Wei Zi has stronger antibacterial activity and concentration-dependence compared with the aqueous extract. WWZE has a MIC value of 1.25 mg/mL against *V. parahaemolyticus*, which is equivalent to that of chitosan [26]. Seven lignans were further identified by HPLC. The free lignans are lipophilic, which also verified the correctness of extracting WWZE by ethanol aqueous solution.

Hakala et al. found that six lignans (schisandrol A, schisandrol B, schisantherin A, and schisandrin A–C) could inhibit *C. pneumoniae* inclusion formation and infectious progeny production. The presence and substitution mode of methylenedioxy, methoxy, and hydroxyl groups in lignans has an important effect on their antichlamydial activity. However, the six lignans had no inhibitory effect on seven other bacteria, indicating that their antibacterial effect had a certain degree of selectivity [27]. Similarly, we found that schisantherin A and schisandrol B were the main anti-*V. parahaemolyticus* active molecules of WWZE, while the other five lignans showed no activities against *V. parahaemolyticus*. In addition, there are other kinds of antibacterial active ingredients in WWZE. Bai et al. found that the antimicrobial activity of Wu Wei Zi was mainly due to organic acids such as citric acid and malic acid [24], which also indicated that the antibacterial properties of WWZE are the result of the synergistic action of various chemical components.

The development of bacterial biofilms can be divided into four stages: initial adhesion, proliferation, maturation, and diffusion [28]. In this study, it was found that when WWZE was added at the adhesion and proliferation stages, it inhibited the biofilm formation, and when added at the mature stage, it had a scavenging effect on the mature biofilm. Both showed a dose-dependent pattern, with inhibition and clearance of 76.12% and 46.28% at 2MIC, respectively, which also confirmed that bacteria in biofilms are much more resistant to drugs than in their planktonic form [29]. Previous studies by Lu et al. [30] found that the inhibition and clearance of cinnamon extract against *V. parahaemolyticus* biofilms at 2MIC were 96.65% and 20.97%, respectively.

Due to the lipophilic nature of WWZE, the cell membrane may be the initial target for its action on *V. parahaemolyticus* [31]. This study found that WWZE at MIC concentration could lead to a significant increase in the extracellular protein content of bacteria in both forming and mature biofilms, indicating that the membrane integrity of *V. parahaemolyticus* was destroyed after treatment with WWZE. This is consistent with the effect of eugenol on antibiotic-resistant *V. parahaemolyticus* [7]. Therefore, WWZE can first penetrate and destroy the cell membrane, resulting in bacterial death. On the other hand, extracellular proteins may provide support for the continued adhesion and aggregation of viable bacteria, contributing to the continued formation of biofilms [32].

Extracellular polymeric substances (EPS) that provide protection for biofilm cells are usually composed of extracellular polysaccharide, eDNA, and protein in varying amounts [28]. As the main component of exopolysaccharides, PIA is a partially deacetylated, positively charged poly-β(1-6)-*N*-acetylglucosamine (PNAG), which has a strong ability to promote adhesion [33]. eDNA is produced by bacteria through active apoptosis or passive autophagy, and plays a role in promoting cell connectivity and stabilizing matrix structure during the cell adhesion stage and biofilm maturation stage, respectively [34]. Therefore, we determined the mechanism of action of WWZE on *V. parahaemolyticus* biofilm. The results showed that WWZE could significantly inhibit the synthesis of PIA on the surface of biofilm and the secretion of eDNA in a dose-dependent pattern even at sub-MIC concentrations, thereby inhibiting the formation of *V. parahaemolyticus* biofilm.

Furthermore, WWZE at MIC concentration reduced the metabolic activity of the forming biofilm and mature biofilm by 81.98% and 33.92%, respectively. On the one hand, it was confirmed that WWZE exerts its anti-*V. parahaemolyticus* biofilm effect by inhibiting the synthesis of PIA and the secretion of eDNA. On the other hand, it was also verified that once pathogenic bacteria form a biofilm, they are more resistant to drugs and quicker to recover from physical removal than planktonic cells [35].

## 4. Materials and Methods

### 4.1. Materials and Reagents

Wu Wei Zi was purchased from Zhendong Pharmacy (Lianyungang, China); *V. parahaemolyticus* 1.1997 was preserved in our laboratory; MH broth (MHB) medium was purchased from Hangzhou Best Biotechnology Co., Ltd.; schisandrol A, schisandrol B, schisantherin A, schisandrin A, schisandrin B, and schisandrin C were obtained from Medesheng Technology Co., Ltd. (Chengdu, China); schisanhenol was purchased from Alfa Biotechnology Co., Ltd. (Chengdu, China); crystal violet staining solution was obtained from Huankai Microbial Technology Co., Ltd. (Hangzhou, China); CCK-8 cell viability detection kit was purchased from Feijing Biotechnology Co., Ltd. (Fuzhou, China); bacterial genomic DNA extraction kit was obtained from Fuji Biotechnology Co., Ltd. (Chengdu, China); Congo red, the chromatographic grade methanol, and other analytical grade solvents were obtained from Sinopharm Chemical Reagent Co., Ltd. (Shanghai, China).

### 4.2. Single-Factor Experiment

A total weight of 1.0 g of Wu Wei Zi fine powder was accurately weighed and put into a 250 mL Erlenmeyer flask, and 20 mL of ethanol solvent of different concentrations (60%, 70%, 80%, 90%, and 100%) was added. The mixtures were extracted in a constant temperature bath at 80 °C for 120 min, and filtered with Büchner funnel. The filtrate was prepared to 20 mL for evaluating the effect of ethanol concentration on the antimicrobial activity of WWZE against *V. parahaemolyticus* by agar diffusion method. Similarly, the effects of extraction temperature (60, 70, 80, 90, and 100 °C), extraction time (60, 90, 120, 150, and 180 min) and liquid–solid ratio (5:1, 10:1, 15:1, 20:1, and 25:1 mL/g) on the antimicrobial activity of WWZE against *V. parahaemolyticus* were measured sequentially.

### 4.3. Box–Behnken Design

Design Expert 8.0 (Stat-Ease, Minneapolis, USA) was used to optimize the extraction conditions using Box–Behnken design (BBD) and response surface methodology (RSM) [36]. Ethanol concentration (*X*_1_), extraction temperature (*X*_2_), and extraction time (*X*_3_) were taken as independent variables of BBD, and the diameter of the inhibitory circle of WWZE against *V. parahaemolyticus* was taken as the response factor (*Y*, mm). The experimental results were analyzed using Design Expert 8.0 software, and the following second-order polynomial equation was used to evaluate the mode of the system:

$$Y=\beta_{0}+\sum_{i=1}^{3}\beta_{i}X_{i}+\sum_{i=1}^{3}\beta_{ii}X_{i}^{2}+\sum_{i=1}^{2}\sum_{j=i+1}^{3}\beta_{ij}X_{i}X_{j}$$

where *Y* is the predicted response value; *β*_0_, *β_i_*, *β_ii_*, and *β_ij_* are the regression coefficients in the intercept, linear, and quadratic terms; *X_i_* and *X_j_* are independent variables.

### 4.4. Agar Diffusion Method

The antibacterial activity of WWZE against *V. parahaemolyticus* were measured by agar diffusion method [37]. A volume of 100 µL bacterial suspension (10^6^ cfu/mL) was uniformly coated on the solidified MHB agar petri dish and the Oxford cups were placed. Thereafter, 200 µL of WWZE was added and incubated at 37 °C for 24 h, then the diameter of bacteriostatic zone was measured with an electronic vernier caliper.

### 4.5. High Performance Liquid Chromatography

Schisandrol A, schisandrol B, schisantherin A, schisandrin A–C, and schisanhenol were used as references to identify the chemical components in WWZE by high-performance liquid chromatography (HPLC) [16]. The determination was performed on a CLC-ODS column (10.0 × 250 mm, 5 μm, 1.5 mL/min) with a detection wavelength of 254 nm. The mobile phases were 0.1% phosphoric acid water and CH_3_OH. The gradient elution procedure followed was (*v*/*v*): 0 min 80% CH_3_OH, 25 min 90% CH_3_OH, 30 min 100% CH_3_OH, 55 min 100% CH_3_OH.

### 4.6. Broth Microdilution Method

The minimum inhibitory concentration (MIC) of WWZE and their main components against *V. parahaemolyticus* was determined by broth microdilution method [28]. Volumes of 2 μL of different concentrations of sample solution dissolved with DMSO were added to a 96-well plate containing 198 μL of bacterial suspension (10^5^ cells/mL), respectively. The control was 2 μL DMSO instead of the sample solution. After being cultured at 37 °C for 24 h, MIC was defined as the lowest concentration at which bacterial growth is not visible to the naked eye.

### 4.7. Crystal Violet Method

The inhibition effects of WWZE on the formation of biofilm and the removal effect of pre-formed biofilm were determined by crystal violet method [38]. The inhibitory effect on biofilm formation was that 2 μL of WWZE solution dissolved with DMSO to a 96-well plate containing 198 μL of bacterial suspension (10^5^ cells/mL) in each well to a final concentration of 2MIC, MIC, 1/2MIC, and 1/4MIC, respectively; The elimination effect on pre-formed biofilms first required forming biofilms by incubating a 96-well plate with 198 μL bacterial suspension (10^5^ cells/mL) in each well at 37 °C for 24 h. Then, 2 μL of WWZE solution dissolved with DMSO to a 96-well plate containing 198 μL of bacterial suspension (10^5^ cells/mL) in each well to a final concentration of 2MIC, MIC, 1/2MIC, and 1/4MIC, respectively. The control was 2 μL DMSO instead of sample solution, incubated at 37 °C for 24 h. The medium was discarded, and the biofilm was washed twice with 200 μL PBS solution and fixed at room temperature for 30 min. The biofilm was stained with 0.1% crystal violet for 20 min, and the liquid was discarded and washed twice with 200 μL PBS solution. The absorbance was measured at 595 nm after 33% acetic acid was dissolved for 30 min.

### 4.8. Coomassie Brilliant Blue Method

The effect of WWZE on the membrane integrity of *V. parahaemolyticus* was determined by Coomassie brilliant blue method [7]. A volume of 2 μL of WWZE solution dissolved with DMSO was added to a 96-well plate containing 198 μL of bacterial suspension (10^5^ cells/mL) or pre-formed biofilm to make the final concentrations of 2MIC, MIC, 1/2MIC, and 1/4MIC, and the control was 2 μL DMSO instead of sample solution and incubated at 37 °C for 24 h. After the incubation, the medium in each well was collected and centrifuged at 4000 r/min for 10 min. A volume of 20 μL of the supernatant was pipetted into the 96-well plate. A volume of 200 μL of Coomassie brilliant blue G-250 was added and left to stand for 5 min, then the absorbance at 600 nm was measured by microplate reader.

### 4.9. Congo Red Plate Method

The effect of WWZE on the synthesis of PIA of *V. parahaemolyticus* was determined by the Congo red plate method [28]. The single colonies of overnight-cultured *V. parahaemolyticus* were inoculated into Congo red plates containing different concentrations of WWZE. The experimental results were observed after 24 h of inverted culture at 37 °C.

### 4.10. Spectrophotometry

The effect of WWZE on eDNA secretion of *V. parahaemolyticus* was determined by spectrophotometry [29]. A volume of 2 μL of WWZE solution dissolved with DMSO was added to a 96-well plate containing 198 μL of bacterial suspension (10^5^ cells/mL) to make the final concentrations of 0, 1/2MIC, MIC, and 2MIC, and the control was 2 μL DMSO instead of sample solution and incubated at 37 °C for 24 h. eDNA was extracted by bacterial genomic DNA extraction kit, and OD_260_/OD_280_ ratio was detected by spectrophotometer.

### 4.11. CCK-8 Method

The effect of WWZE on the metabolic activity of *V. parahaemolyticus* biofilm was determined by CCK-8 method [39]. A volume of 2 μL of WWZE solution dissolved with DMSO was added to a 96-well plate containing 198 μL of bacterial suspension (10^5^ cells/mL) or pre-formed biofilm to make the final concentrations of 2MIC, MIC, ½MIC, and 1/4MIC, and the control replaced the sample solution with 2 μL DMSO and incubated at 37 °C for 24 h. After the incubation, the cells were washed twice with 200 μL PBS to remove planktonic and loosely attached cells after the medium was discarded. A volume of 180 μL of PBS and 20 μL of CCK-8 solution were added into each well and cultured at 37 °C for 4 h. The absorbance at 600 nm was measured after being mixed well.

### 4.12. Statistical Analysis

All assays were performed for three times, and the results were expressed by mean ± standard deviation. The difference significance test was conducted by Student’s t-test with *p* values < 0.05 considered significant.

## 5. Conclusions

In conclusion, WWZE can be used as an anti-*V. parahaemolyticus* agent and the optimal extraction conditions for WWZE were ethanol concentration of 69%, temperature of 91 °C, time of 143 min, and liquid–solid ratio of 20:1 mL/g. The MIC of WWZE against *V. parahaemolyticus* is 1.25 mg/mL, which is a synergistic effect of various chemical components such as schisantherin A and schisandrol B with the MIC values of 0.625 and 1.25 mg/mL, respectively. MIC concentration of WWZE can effectively inhibit biofilm formation and clear mature biofilm of *V. parahaemolyticus* up to 67.69% and 34.17% by significantly destroying cell membrane integrity, inhibiting PIA synthesis and eDNA release, and reducing metabolic activity of biofilm. The study reported for the first time the favorable anti-biofilm effect of WWZE against *V. parahaemolyticus*, which provides a basis for deepening the application of WWZE in aquatic product preservation. Therefore, this study demonstrates the potential of WWZE to reduce pathogen contamination as a natural food preservative.

## Figures and Tables

**Table 1:** Experimental design and response values.

No.	*X*_1_	*X*_2_	*X*_3_	*Y*
	Ethanol Concentration (%)	Temperature (°C)	Time (min)	Diameter of Inhibitory Circle (mm)
1	−1 (60)	−1 (80)	0 (150)	11.36
2	+1 (80)	−1 (80)	0 (150)	13.09
3	−1 (60)	+1 (100)	0 (150)	12.99
4	+1 (80)	+1 (100)	0 (150)	12.14
5	−1 (60)	0 (90)	−1 (120)	14.06
6	+1 (80)	0 (90)	−1 (120)	12.64
7	−1 (60)	0 (90)	+1 (180)	12.46
8	+1 (80)	0 (90)	+1 (180)	12.78
9	0 (70)	−1 (80)	−1 (120)	12.03
10	0 (70)	+1 (100)	−1 (120)	13.93
11	0 (70)	−1 (80)	+1 (180)	11.99
12	0 (70)	+1 (100)	+1 (180)	11.95
13	0 (70)	0 (90)	0 (150)	15.69
14	0 (70)	0 (90)	0 (150)	15.21
15	0 (70)	0 (90)	0 (150)	15.45
16	0 (70)	0 (90)	0 (150)	15.09

**Table 2:** Analysis of variance on the effect of ethanol concentration, extraction temperature, and extraction time on the diameter of the inhibitory circle of WWZE.

Source	Sum of Squares	df	Mean Square	F Value	Prob > F	Sig.
Model	32.57	9	3.62	27.32	0.0001	**
*X*_1_	0.00605	1	0.00605	0.05	0.8369	
*X*_2_	0.81	1	0.81	6.09	0.0430	*
*X*_3_	1.51	1	1.51	11.43	0.0117	*
*X*_1_ *X*_2_	1.66	1	1.66	12.56	0.0094	**
*X*_1_ *X*_3_	0.76	1	0.76	5.71	0.0481	*
*X*_2_ *X*_3_	0.94	1	0.94	7.10	0.0322	*
*X*_1_^2^	6.22	1	6.22	46.96	0.0002	**
*X*_2_^2^	12.54	1	12.54	94.64	<0.0001	**
*X*_3_^2^	5.43	1	5.43	40.99	0.0004	**
Lack of Fit	0.70	3	0.23	4.19	0.1001	

* *p* < 0.05, ** *p* < 0.01.

## Data Availability

Not applicable.

## Sample Availability

Samples of the compounds WWZE are available from the authors.
